# Draft Genome Sequence of the Capripoxvirus Vaccine Strain KSGP 0240, Reisolated from Cattle

**DOI:** 10.1128/MRA.00440-21

**Published:** 2021-07-29

**Authors:** Zahra Bamouh, Siham Fellahi, Slimane Khayi, Jihane Hamdi, Khalid Omari Tadlaoui, Ouafaa Fassi-Fihri, Mehdi Elharrak

**Affiliations:** a Research and Development Department, Multi-Chemical Industry, Mohammedia, Morocco; b Institute of Agronomy and Veterinary Medicine Hassan II, Rabat, Morocco; c CRRA-Rabat, National Institute for Agricultural Research (INRA), Rabat, Morocco; Portland State University

## Abstract

Control of lumpy skin disease in cattle is based on vaccination with live attenuated vaccines. The Kenyan strain KSGP 0240 is commonly used to vaccinate ruminants against capripox infections, but the conferred protection is still controversial. In this study, we report the draft genome sequence of the vaccine strain KSGP 0240, reisolated from cattle.

## ANNOUNCEMENT

Lumpy skin disease is an emerging disease of cattle throughout Africa, the Middle East, and Central Asia, with high economic impact (1). Lumpy skin disease is caused by a virus from the family *Poxviridae*, genus *Capripoxvirus*. Vaccination plays a key role to control the spread of the disease in regions of endemicity.

The Kenyan vaccine strain KSGP 0240, of cattle origin, is commonly used to vaccinate sheep/goats and cattle against capripox infections (2, 3). However, Ayelet et al. (3) and Tuppurainen et al. (4) reported that the vaccine based on this strain did not confer the expected protection (3, 4), probably because the strain was adapted to growth on a heterologous system (Vero cells), which limited its capability to replicate on the target species (5). Strain KSGP 0240 was provided by the Pirbright Institute. In order to refresh the virus on a homologous system, we carried out one passage on cattle. Five milliliters of a viral suspension (10^5.0^ 50% tissue culture infective doses [TCID_50_]/ml) obtained from primary testis cells infected with KSGP 0240 was injected subcutaneously into a 6-month-old naive calf. After 10 days of incubation, the virus was recovered aseptically from cells of the homogenate taken from inflammatory tissue at the injection site, after centrifugation at 3,000 × *g* for 20 min (6).

The virus from the calf was repassaged on testis cells before DNA isolation; 200 ng of genomic DNA was extracted using the Isolate II genomic DNA kit (Qiagen) according to the manufacturer’s instructions (7). The whole-genome sequencing was outsourced to Eurofins Genomics Company, using the Illumina NovaSeq 6000 platform in 2 × 150-bp paired-end mode. The library construction protocol used was validated and established by the company on the basis of NEBNext Ultra II directional DNA library prep kit for Illumina using TruSeq adapter sequences. The quality of the final library in terms of size and concentration was assessed by the company.

Prior to the assembly, 14,283,922 raw reads with an average length of 151 bp were trimmed using CLC Genomics Workbench v12 (Qiagen) (parameters: limit = 0.05, ambiguous nucleotides *n* ≤ 2). In total, 14,283,800 trimmed reads were then *de novo* assembled using CLC Genomics Workbench v12. The viral genome contains a contig comprising 146,090 bp and a G+C content of 26%, with an average coverage of 4,000×. The trimmed reads were mapped against the reference sequence (GenBank accession number KX683219) with a length fraction = 0.5 and similarity fraction = 0.8 as the parameters; then, the total reads mapped (4,134,790 reads) were used for variant calling using CLC Genomics Workbench. The analysis revealed seven variants: two single-nucleotide deletions, three single-nucleotide substitutions (SNV), and two single-nucleotide insertions. Consequently, three genes were affected: the hypothetical protein gene LSD026, the mRNA capping enzyme small subunit gene LSD089, and the putative virion core protein gene LSD094 (Table 1). The sequence of the vaccine strain passaged in cattle is 99.99% identical to the reference sequence (KX683219) based on BLASTN analysis (5).

**TABLE 1:** Single-nucleotide polymorphisms of vaccine strain KSGP 0240 after passage

Reference position	Type	Length^*a*^ (bp)	Reference	Allele	Count^*b*^	Coverage^*c*^ (×)	Frequency (%)	Overlapping annotations^*d*^	Average quality of variant^*e*^	Coding region change	Amino acid change
17	SNV	1	T	A	7,705	7,760	99.29		35.53		
18578	Deletion	1	A		3,167	3,335	94.96	Gene: LSDV026; CDS: LSDV026	35.73	AOE47602.1:c.479del	AOE47602.1:p.Leu160fs
22772	Insertion	1		A	3,147	3,437	91.56		36.39		
28073	Insertion	1		T	1,806	3,916	46.12		35.85		
84168	SNV	1	C	A	3,457	3,486	99.17	Gene: LSDV089; CDS: LSDV089	36.6	AOE47665.1:c.48G > T	AOE47665.1:p.Leu16Phe
89076	SNV	1	G	T	3,881	3,886	99.87	Gene: LSDV094; CDS: LSDV094	36.11	AOE47670.1:c.254C > A	AOE47670.1:p.Pro85His
125083	Deletion	1	A		3,771	4,119	91.55		33.62		

a Number of nucleotides comprised by the variants.

b Number of reads mapped to the position, including the variants.

c Total number of reads mapped onto the variants.

d CDS, coding DNA sequence.

e Average read quality score of the bases supporting a variant.

### Data availability.

This whole-genome shotgun project has been deposited at DDBJ/ENA/GenBank under the accession number MW631933. The version described in this paper is version MW631933. The Illumina reads are available in the SRA under the accession number SRX10508754.
